# PSMA-positive nodal recurrence in prostate cancer: Salvage radiotherapy is superior to salvage lymph node dissection in retrospective analysis

**DOI:** 10.1007/s00066-020-01605-z

**Published:** 2020-03-24

**Authors:** Nina-Sophie Schmidt-Hegemann, Alexander Buchner, Chukwuka Eze, Paul Rogowski, Christian Schaefer, Harun Ilhan, Minglun Li, Wolfgang Peter Fendler, Peter Bartenstein, Ute Ganswindt, Christian Stief, Claus Belka, Alexander Kretschmer

**Affiliations:** 1 Department of Radiation Oncology, University Hospital, LMU Munich, Marchioninistr. 15, 81377 Munich, Germany; 2 Department of Urology, University Hospital, LMU Munich, Marchioninistr. 15, 81377 Munich, Germany; 3 Department of Nuclear Medicine, University Hospital, LMU Munich, Marchioninistr. 15, 81377 Munich, Germany; 4 Department of Nuclear Medicine, University Hospital Essen, Hufelandstr. 55, 45147 Essen, Germany; 5 Department of Therapeutic Radiology and Oncology, Innsbruck Medical University, Anichstr. 35, 6020 Innsbruck, Austria; 6 German Cancer Consortium (DKTK), Munich, Germany

**Keywords:** Prostate cancer, PSMA PET/CT, Biochemical recurrence, Biochemical persistence, Radiotherapy, Salvage lymph node dissection

## Abstract

**Purpose:**

This analysis compares salvage lymph node dissection (SLND) to salvage lymph node radiotherapy (SLNRT) of ^68^Ga-PSMA PET-positive nodal recurrences after radical prostatectomy (RPE).

**Methods:**

A total of 67 SLNRT and 33 SLND consecutive patients with pelvic and/or para-aortic nodal recurrences after RPE were retrospectively analyzed. Biochemical recurrence-free survival rates (bRFS; PSA <0.2 ng/mL) were calculated according to Kaplan–Meier and survival curves were compared using the log rank test. For multivariable analysis, binary logistic regression analysis was performed (*p* < 0.05).

**Results:**

Median follow-up was 17 months (range, 6–53 months) in SLND patients and 31 months (range, 3–56 months) in SLNRT patients (*p* = 0.027). SLNRT patients had significantly more tumours of pT3 and pT4 category (82% vs. 67%; *p* = 0.006), pathologically involved lymph nodes (45% vs. 27%; *p* = 0.001) and positive surgical margins (54% vs. 12%; *p* = 0.001) at time of RPE than SLND patients. PSA persistence after RPE was significantly more frequently observed in the SLNRT cohort (73% vs. 27%; *p* = 0.001). There was no significant difference in the distribution of PET-positive lymph nodes. Median PSA before SLND was higher than before SLNRT (3.07 ng/ml vs. 1.3 ng/ml; *p* = 0.393). The 2‑year bRFS was significantly higher in the SLNRT vs. the SLND cohort (92% vs. 30%; *p* = 0.001) with lower rates of distant metastases (21% vs. 52%; *p* = 0.002) and secondary treatments (5% vs. 39%; *p* = 0.011) irrespective of ongoing androgen deprivation therapy at last contact. In multivariable analysis, SLNRT was significantly associated with prolonged bRFS (regression coefficient 1.436, hazard ratio 4.204, 95% CI 1.789–9.878; *p* = 0.001).

**Conclusion:**

Based on this retrospective study SLNRT might be the preferred treatment option for patients with nodal recurrence after previous RPE.

**Electronic supplementary material:**

The online version of this article (10.1007/s00066-020-01605-z) contains supplementary material, which is available to authorized users.

## Introduction

Up to 30% of patients with high-risk prostate cancer (PCa) relapse after radical prostatectomy (RPE) with the majority of patients having lymph node recurrences [1–3]. Management of lymph node recurrence after RPE is a challenging clinical scenario, most times involving different specialists. Salvage lymph node dissection (SLND) and salvage lymph node radiotherapy (SLNRT) are the two possible treatment options for metastasis-directed therapy (MDT) of node-positive PCa. Even though SLND and SLNRT are regularly performed, there is sparse comparative data regarding beneficial effects on survival outcomes.

In a recent systematic review on SLND, wide ranges of 2‑ and 5‑year biochemical recurrence-free survival rates (bRFS) between 23 to 64% and 6 to 31%, respectively, have been described. In addition, the 5‑year overall survival was about 84% [4]. However, it has to be stated that templates of SLND varied significantly between the currently available studies, as did adjuvant treatment, endpoints, and study populations, and present evidence is mostly based on small single-centre case series [4].

Likewise, few studies on SLNRT were identified in a recent review: Patients with pelvic and/or extra-pelvic nodes were either treated by stereotactic body radiotherapy (SBRT) (55%) or elective nodal radiotherapy (ENRT) (45%) to a lymph node region or the whole pelvic lymphatic pathways [5]. In patients treated with ENRT 3‑year progression-free survival ranged from 61.8 to 75% [6], whereas in patients with SBRT to PET-positive lymph nodes 3‑year progression-free survival ranged between 26 and 33% [5]. This is strikingly comparable to outcome data achieved by SLND, although one has to reconsider that the majority of patients in the reported studies were staged at best with choline positron emission tomography/computer tomography (PET/CT) thus underestimating the true extent of nodal recurrence.

Hereby, a significant benefit for ^68^gallium-prostate-specific membrane antigen PET/CT (^68^Ga-PSMA PET/CT) was observed, and PSMA-PET/CT has since then evolved towards the new imaging reference standard with a high detection rate of lymph nodes in case of PSA persistence or recurrence [7–11]. This might improve outcome by enabling a more extended lymphadenectomy or a higher dose administration to PET-positive lymph nodes and at the same time a more comprehensive ENRT.

Thus, this current analysis was restricted to patients with PSMA PET-positive lymph node recurrences after RPE in order to compare SLND to SLNRT at a tertiary care centre.

## Materials and methods

### Patient population

Between February 2014 and December 2018, 100 consecutive patients underwent ^68^Ga-PSMA PET/CT prior to SLNRT (67 patients) or SLND (33 patients) with evidence of PET-positive pelvic and/or para-aortic lymph nodes after RPE. Patients who underwent previous adjuvant radiotherapy of the pelvic lymphatic pathways or ADT prior to SLNRT/SLND were excluded. Patients were subgrouped according to the D’Amico criteria [12] incorporating tumour-stage, PSA level and Gleason score (GS) (Table 1). All patients provided written informed consent to undergo ^68^Ga-PSMA PET/CT. This retrospective analysis was performed in compliance with the principles of the Declaration of Helsinki and its subsequent amendments [13].

**Table 1:** Patients’ characteristics

	SLND	SLNRT	*p*-value
**Patients**	*n* = 33	*n* = 67	
Age (years) median (range) at time of LN dissection/RT	66 (52–83)	72 (47–83)	0.151
Initial PSA at RPE (ng/ml) median (range)	10.40 (2.01–262)	17.30 (3.99–190)	0.334
**Gleason score**^a^			0.059
≤6–7	20 (61%)	28 (42%)	
8–9	13 (39%)	39 (58%)	
**TNM at time of RPE**			
*T‑stage*			0.006
T2	11 (33%)	12 (18%)	
T3–4	22 (67%)	55 (82%)	
*N‑stage*			0.001
N0	17 (52%)	36 (54%)	
N1	9 (27%)	30 (45%)	
N x	7 (21%)	1 (1%)	
Number of removed LN median (range)	11 (4–37)	14 (2–45)	
Number of positive LN median (range)	0 (0–5)	0 (0–16)	
M0	33 (100%)	67 (100%)	
Positive surgical margins	4 (12%)	36 (54%)	0.001
**D’Amico classification**			0.967
Low	1 (3%)	2 (3%)	
Intermediate	5 (15%)	5 (7%)	
High	27 (82%)	60 (90%)	
**Postoperative PSA median (range)**	0.30 (<0.03–10)	0.59 (<0.03–58)	0.642
**PSA persistence/recurrence**			0.001
Number of patients with PSA persistence	9 (27%)	49 (73%)	
Number of patients with PSA recurrence	24 (73%)	18 (27%)	
**PSA at PET (ng/ml) median (range)**			
PSA persistence	1.75 (0.21–19.50)	1.60 (0.14–40.13)	0.391
PSA recurrence	1.70 (0.30–16.0)	0.67 (0.31–6.76)	0.342
**PSMA PET/CT result**			0.001
Lymph node positive only	33 (100%)	51 (76%)	
Fossa and lymph node recurrence	0 (0%)	16 (24%)	
Number of PET-positive lymph nodes (median; range)	1 (1–10)	2 (1–19)	0.392
**Distribution of suspect lymph nodes**			0.063
Pelvic	25 (76%)	54 (81%)	
Retroperitoneal	4 (12%)	1 (1%)	
Both	4 (12%)	12 (18%)	
**Time (months) between RPE and SLN dissection/RT start median (range)**			0.941
All patients	9 (2–120)	6 (2–194)	
PSA persistence	7 (2–84)	5 (2–86)	
PSA recurrence	11 (2–120)	69 (9–194)	

*SLND* salvage lymph node dissection, *SLNRT* salvage lymph node radiotherapy, *PSA* prostate specific antigen, *RPE* radical prostatectomy, *LN* lymph node, *RT* radiotherapy

^a^Data on Gleason score obtained from radical prostatectomy

### ^68^Ga-PSMA labelling and PET/CT imaging

Radiolabelling of PSMA-HBED-CC was performed with ^68^Ga^3+^ from a ^68^Ge/^68^Ga generator system (GalliaPharm®, Eckert & Ziegler AG, Berlin, Germany) using an automated synthesis module (GRP, Scintomics GmbH, Munich, Germany) and prepacked cassettes (ABX GmbH, Radeberg, Germany) as described previously for a different PSMA ligand by Weineisen et al. [14]. ^68^Ga-PSMA PET/CT imaging was performed according to current guidelines [15] with a Siemens Biograph 64 or GE Discovery 690 PET/CT camera. Phantom studies based on the National Electrical Manufacturers Association NU2-2001 standard were conducted to allow valid pooling of the results, and standardized uptake value (SUV) conversion factors were calculated [16]. ^68^Ga-PSMA PET/CT scans were performed with a diagnostic CT scan (reference mAs, 200–240; 120 kV) and obtained with intravenous injection of iodine-containing contrast agent (Ultravist 300, Bayer Pharma AG, Berlin, Germany; or Imeron 300, Bracco, Konstanz; 2.5 mL/s; in portal venous phase) around 60 min after intravenous administration of ^68^Ga-PSMA. In absence of contraindications, 20 mg furosemide was injected almost simultaneously with ^68^Ga-PSMA injection to lower tracer retention in the bladder. PET images were reconstructed with an axial 168 × 168 matrix based on the TrueX algorithm (3 iterations, 21 subsets; Biograph 64) or on the VUE Point FX algorithm (2 iterations, 36 subsets; Discovery 690).

### Image interpretation

PET/CT images were interpreted by a consensus read of two nuclear medicine physicians and two radiologists in the sense of a clinical report-based analysis. At least two of the readers had more than 10 years PET/CT experience. The location of each lesion was determined by CT. PET-positive lesions were visually identified by ^68^Ga-PSMA uptake above background level and not associated with physiologic uptake [15].

### Radiotherapy

All patients received ENRT by intensity-modulated radiotherapy (IMRT) or volumetric arc therapy (VMAT) and image-guided radiotherapy techniques (IGRT, 2–5 times/week). Radiotherapy dose regimens were normo- or slightly hypofractionated and a boost to the PET-positive local recurrences within prostatic fossa and lymph nodes was applied simultaneously. The prostatic fossa was treated with a median of 66 Gy (range, 60–67.20 Gy) in single doses of 2 Gy (range, 1.8–2.12 Gy) and the elective lymphatic pathways with 50.4 Gy (range, 45–52.28 Gy) in single doses of 1.8 Gy (range, 1.6–1.8 Gy). PSMA PET-positive local recurrences within in the prostatic fossa were irradiated with 70.0 Gy (range, 68–70 Gy) and PSMA PET-positive lymph nodes with 61.6 Gy (range, 50.4–66 Gy). Planning CT was done in supine position with 3 mm slice thickness. Patients were advised to have a full bladder and empty rectum. Target delineation was performed according to the Radiation Therapy Oncology Group (RTOG) atlas for salvage PCa [17] and for lymphatic pathway delineation [18].

### Lymph node dissection

The standard SLND procedures at our department have been described before [7, 19]. Briefly, an open approach through an abdominal midline incision was used and standard extended SLND was performed based on specific regions according to the most recent PSMA PET findings. Dissected lymph nodes were classified based on the respective anatomic region. Routinely, dissected lymph nodes were immediately sent for histopathologic analysis and evaluated according to standard protocols (serial sectioning, 200 μm slices).

### Treatment application and follow-up

Treatment management following PSMA PET was documented for each patient. Follow-up time was defined as the interval in months between SLND/SLNRT and the last recorded PSA. Follow-up examination was first performed 6 weeks to 3 months after SLND/SLNRT and then every 6–12 months. Patients were regarded free of ADT influence after a minimum time-period of 5 months after last application of ADT.

### Statistical analysis

BRFS (PSA ≤0.2 ng/ml) was defined as the primary study endpoint. For statistical analysis, SPSS Statistics 25 (IBM, New York, NY, USA) was used. Demographic data were analysed using descriptive statistics and χ^2^ test. Time to event data was calculated using the Kaplan–Meier method. Differences between subgroups were compared using log rank test with a *p* value of <0.05 considered statistically significant. The χ^2^ test, Mann–Whitney U test and binary logistic regression were performed to determine the influence of SLND vs. SLNRT, GS ≤6–7 vs. GS 8–9, tumour stage T2 vs. T3–4, nodal stage N0/N x vs. N1, D’Amico stage low/intermediate vs. high, PSA at salvage therapy, PSA persistence vs. PSA recurrence and PET-positive pelvic vs. pelvic and/or retroperitoneal lymph nodes on bRFS.

## Results

### Patients’ characteristics and PSMA PET results

Most patients (87/100; 87%) had a high-risk PCa at time of RPE with no significant difference between the SLND and SLNRT cohort. SLNRT patients had significantly more tumours with locally advanced pT3 or pT4 disease (82% vs. 67%; *p* = 0.006) compared to the SLND cohort with significantly more tumours of pT2 category (33% vs. 18%). Furthermore, there were significantly more patients in the SLNRT cohort with evidence of pathologically involved lymph nodes (45% vs. 27%; *p* = 0.001) and positive surgical margins (54% vs. 12%; *p* = 0.001) at time of RPE. Overall, there were 58 patients with PSA persistence and 42 patients with PSA recurrence after RPE. SLNRT for PET-positive lymph nodes was significantly more often applied in patients with PSA persistence (73%) than PSA recurrence (27%). On the other hand, SLND was performed significantly more often in patients with PSA recurrence (73%) than PSA persistence (27%; *p* = 0.001) (Table 1). Time between RPE and SLND/SLNRT was longer in patients with PSA recurrence than PSA persistence (6 vs. 44 months and 5 vs. 69 months). Patients with SLND had only evidence of PET-positive lymph nodes (100%). Patients with SLNRT had PET-positive lymph nodes with (24%) or without (76%) local recurrence within prostatic fossa (*p* = 0.001). There was no significant difference in the distribution of PET-positive lymph nodes between the two cohorts: Only few patients had evidence of para-aortic lymph nodes (24% in the SLND group and 19% in the SLNRT group; *p* = 0.063).

### Management of PET-positive lymph nodes and toxicity

Median PSA prior SLND was higher than prior SLNRT (3.07 ng/ml vs. 1.3 ng/ml; *p* = 0.393). Overall, a median of 8 (1–44) lymph nodes were removed at time of SLND. Evidence of PCa was pathologically confirmed in a median of 1 (0–16) lymph node. In total, there were 4 patients with a Clavien Grade II (2 × paralytic ileus, 2 × lymphorrhea) and 2 patients with a Clavien Grade IIIa (2 × pulmonary artery embolism) complication (Table 2).

**Table 2:** Treatment characteristics of salvage lymph node dissection cohort

	SLND
*Median PSA before LN dissection*	3.07 (0.26–19.50)
Number of removed LN median (range)	8 (1–44)
Number of positive LN median (range)	1.0 (0–16)
*Complications*	
Intraoperative	1 (3%)
Postoperative	6 (18%)
Clavien	4 × Clavien II, 2 × Clavien IIIa

*SLND* salvage lymph node dissection, *LN* lymph node

ADT was recommended to all patients with SLNRT due to the evidence of PET-positive lymph nodes for 2 years [20, 21]. Consequently, 59/67 (88%) patients were started on ADT before initiation of SLNRT, 42/67 patients (63%) discontinued after a median time of 7 (2–41) months due to patients’ preferences. Median time between end of ADT and last follow-up was 27 months (range 0–48) in those patients with discontinued ADT. In all, 8/67 (12%) patients refused ADT. Patients received radiotherapy treatment as depicted above. Acute grade 2 gastrointestinal and urogenital toxicity were each observed in 19/67 (28%) patients consisting primarily of diarrhoea and increased urinary frequency. Acute grade 3 urogenital toxicity occurred in 1/67 (2%) patients with evidence of urethral stenosis. Late grade 2 toxicity was overall seen in 24/67 (36%) patients with mainly signs of erectile dysfunction and increased urinary frequency. Late grade 3 toxicity was present in 25/67 (37%) patients with mainly erectile dysfunction (36%) (Table 3).

**Table 3:** Treatment characteristics of radiotherapy cohort

	**SLNRT**					
**Median PSA before LN RT**	1.3 (0.10–40.13)					
**RT (Gy/median; range)**						
Former prostate	66 (60–67.2)					
Lymphatic pathways	50.4 (45.0–52.28)					
PET-positive local recurrence	70 (68–70)					
PET-positive LN	61.6 (50.4–66)					
**RT technique**						
VMAT/IMRT & IGRT	67 (100%)					
**ADT with RT**	59 (88%)					
ADT with stop before last follow-up/median duration (months; range)	42 (63%) 7 (2–41)					
Ongoing ADT at last follow-up	17 (25%)					
No ADT	8 (12%)					
**Toxicity**	**Acute toxicity**			**Late toxicity**		
	***n*** **(%)**			***n*** **(%)**		
	**Grade 2**	**Grade 3**	**Grade 4**	**Grade 2**	**Grade 3**	**Grade 4**
**GU**	19 (28%)	1 (2%)	–	17 (25%)	1 (2%)	–
**GI**	19 (28%)	–	–	1 (2%)	–	–
**Other (erectile dysfunction)**				6 (9%)	24 (36%)	

*SLNRT* salvage lymph node radiotherapy, *ADT* androgen deprivation therapy, *RT* radiation therapy, *VMAT* volumetric modulated arc therapy, *IMRT* intensity-modulated radiotherapy, *IGRT* image-guided radiotherapy, *LN* lymph node, *GU* genitourinary, *GI* gastrointestinal

### Patient outcomes

**Fig. 1:**
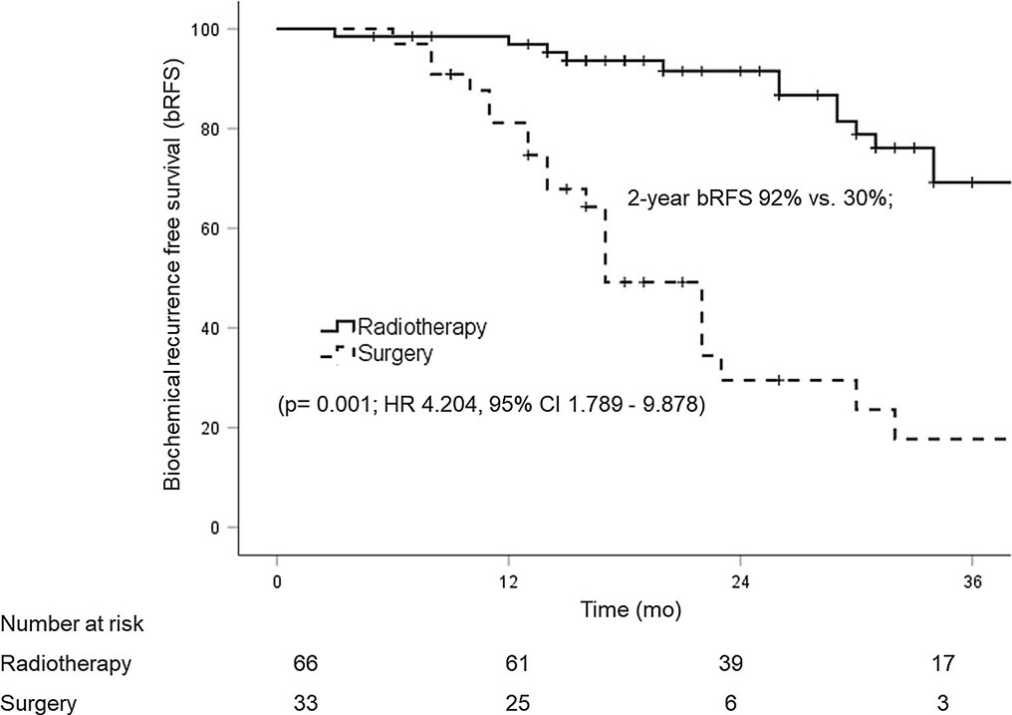
Biochemical recurrence-free survival in patients with salvage lymph node dissection (SLND) vs. salvage lymph node radiotherapy (SLNRT) after metastasis-directed therapy (MDT). *mo* months, *HR* hazard ratio, *95% CI* 95% confidence interval

Median follow-up was 17 months (range, 6–53 months) in patients with SLND and 31 months (range, 3–56 months) in patients with SLNRT (*p* = 0.027). Median posttreatment PSA was significantly higher in patients with SLND (0.47 ng/ml, range <0.03–9.61 ng/ml) than in patients with SLNRT (0.05 ng/ml, range <0.03–19 ng/ml) (*p* = 0.003). At last follow-up, median PSA was 2.50 ng/ml (range <0.03–68 ng/ml) in patients with SLND and 0.05 ng/ml (range <0.03–268 ng/ml) in patients with SLNRT (*p* = 0.025). Overall, the 2‑year bRFS was significantly higher in the SLNRT cohort compared to the SLND cohort (92% vs. 30%; *p* = 0.001) irrespective of ongoing androgen deprivation therapy at last contact (Fig. 1; Table 4). This resulted in a significantly higher rate of distant metastases (52% vs. 21%; *p* = 0.002) and secondary treatments (39% vs. 15%; *p* = 0.011) in patients with SLND. There was one tumour-related death in the SLNRT group.

**Table 4:** Outcome after salvage lymph node dissection/salvage lymph node radiotherapy

	SLND cohort (*n* = 33)	SLNRT cohort (*n* = 67)	*p*-value
*Median follow-up* (months)	17 (6–53)	31 (3–56)	0.027
*Posttreatment PSA *(ng/ml)	0.47 (<0.03–9.61)	0.05 (<0.03–19.0)	0.003
*PSA at last follow-up (ng/ml)*			
Median PSA (range)	2.50 (<0.03–68.0)	0.05 (<0.03–268)	0.025
PSA ≤0.1 ng/ml	7 (21%)	46 (69%)	0.001
PSA ≤0.2 ng/ml	10 (30%)	50 (75%)	0.001
*PSA at last follow-up without ADT (ng/ml)*	*n* = 25	*n* = 42	
Median PSA	1.21 (<0.03–13.0)	0.06 (<0.03–268)	0.02
PSA ≤0.1 ng/ml	5 (20%)	32 (76%)	0.001
PSA ≤0.2 ng/ml	7 (28%)	34 (81%)	0.001
*Secondary treatment (ADT, RT, sx)*	13 (39%)	10 (15%)	0.011
*Clinical progress*			
Distant metastases	17 (52%)	14 (21%)	0.002
*Death*	0	3	0.549
*Tumour-related*	0	1	1.0

*SLND* salvage lymph node dissection, *SLNRT* salvage lymph node radiotherapy, *ADT* androgen deprivation therapy, *PSA* prostate specific antigen, *RT* radiotherapy, *sx* surgery

In multivariable analysis stratified for Gleason score at RPE and PSA prior to PSMA PET/CT, SLND vs. SLNRT was significantly associated with bRFS (regression coefficient 1.473, hazard ratio 4.360, 95% CI 1.665–11.418; *p* = 0.003) (Table 5 and Supplemental Table 1).

**Table 5:** Multivariate analysis of factors associated with biochemical recurrence-free survival after salvage lymph node dissection/salvage lymph node radiotherapy

Association with BRFS (<0.2 ng/mL)	Regression coefficient	HR	95% CI	*p*-Value^a^
SLND vs. SLNRT	1.473	4.360	1.665–11.418	0.003
GS ≤6–7 vs. GS 8–9	1.063	0.345	0.135–0.883	0.027
*PSA at salvage therapy [ng/ml]*	*0.064*	*1.066*	*0.991–1.147*	*0.087*

^a^Binary logistic regression

*SLND* salvage lymph node dissection, *SLNRT* salvage lymph node radiotherapy, *BRFS* biochemical recurrence-free survival, *GS* Gleason Score, *PSA* prostate specific antigen, *HR* hazard ratio, *95% CI* 95% confidence interval

## Discussion

Management of lymph node recurrence after RPE is a challenging clinical scenario, and there is sparse comparative data regarding the beneficial effects of SLND and SLNRT on survival outcomes. Integration of existing evidence is further hampered by differing patient characteristics, diagnostic modalities and therapy sequences [4]. Overall, it is hypothesized that MDT to lymph node recurrences optimizes the locoregional control, possibly limits the risk of distant progression and thereby might improve cancer-specific survival, as has been described by mainly retrospective data [22–26].

In the current study, bRFS of a contemporary patient cohort diagnosed with PSMA PET-positive nodal recurrence of PCa after RPE is provided. To further homogenize the present patient cohort, none of the included patients had ADT or adjuvant radiotherapy of the pelvic lymphatic pathways prior to SLND/SLNRT. In total, 100 patients (*n* = 33 SLND, *n* = 67 SLNRT) were included in the final analysis, of whom all SLNRT patients were treated with ENRT with simultaneous integrated boost (SIB) to the PET-positive lymph nodes. All surgically treated patients received a standard extended SLND.

Regarding the patient characteristics, there were some noticeable differences between the SLND and the SLNRT subgroup. First, patients undergoing SLNRT had a significantly higher proportion of nonorgan confined disease as well as positive surgical margins at the time of RPE compared to SLND patients. As expected, patients with SLND were primarily patients with PSA recurrence (73%), whereas the SLNRT cohort encompassed mostly patients with biochemical persistence (73%). Finally, based on GS score, there was a trend towards more high-risk patients in the SLNRT patient cohort without reaching statistical significance. Even though relevant differences between the two patient subgroups cannot be overlooked, these patients represent typical contemporary patient cohorts in tertiary care centres. Hereby, the SLNRT cohort had overall worse inherent features compared to the SLND cohort. Nevertheless, bRFS was significantly longer in the SLNRT cohort compared to the SLND cohort irrespective of ongoing ADT at the timing of last follow-up. Furthermore, patients of the SLND cohort had a significantly higher rate of distant metastases and need of secondary treatments.

Rischke et al. reported on a similar analysis of 93 patients with exclusively nodal PCa relapse, but staged with choline PET/CT who were either treated by SLND alone (46 patients) or SLND followed by RT (47 patients) to the regions with proven lymph node metastases [27]: Additional RT led to a significant delayed relapse within the treated region (5-year relapse-free rate 70.7% vs. 26.3%), while time to next relapse outside the treated region was almost equal (median 27 months vs. 29.6 months). Furthermore, patients treated with both modalities had significantly lower rates of new recurrent pelvic lymph node metastases compared to patients with surgery only (13% vs. 57.6%). Although, staged with choline PET/CT, this study shows that with current imaging possibilities patients seem to profit from a more extensive therapeutic approach, like a more extended SLND or ENRT.

When one compares the present SLND cohort to groups who studied explicitly patients staged with PSMA PET/CT, one-year progression-free survival (PFS) ranged from 23 to 64%, with higher PFS found in patients with a radio-guided SLND approach [4, 28–30] indicating that this might further allow the dissection of affected lymph nodes that had not been visualized beforehand on PET/CT. Overall, bRFS of the present SLND cohort compares nicely to this range and similar PFS ranges are known for SBRT cohorts [5].

This proportionally lower bRFS rate of the current patient cohort with SLND is comparable to a recent retrospective, multicentre analysis on SBRT vs. ENRT [31]: ENRT was associated with significantly better 3‑year metastasis-free survival (77% vs. 68%) and significantly fewer individuals with local progression (9 vs. 50 patients) compared to SBRT. Like non-extensive SLND, SBRT treats only the PET-positive lymph nodes, whereas ENRT, as it was performed in the current cohort, not only treats the PET-positive, affected nodes, but the whole lymphatic drainage, for instance the entire pelvic lymphatic pathway as well as in general the prostatic fossa especially in patients with locally advanced disease or positive surgical margins. Congruent with the study of Rischke et al. [27], it was further seen that patients following SBRT tend to relapse more often particularly in the pelvic lymph nodes. These findings suggest again that the current imaging modalities are not yet sensitive enough for a restricted node-based surgical or radiotherapy approach [32].

Based on lymph node recurrences detected by choline PET/CT following primary treatment for PCa, De Bruycker et al. described the anatomic patterns of nodal oligorecurrent PCa in relation to different surgical (limited, standard, superextended SLND) and radiotherapy templates. Correspondingly, they found that with ENRT more patients were theoretically fully covered (*p* < 0.02) and the total number of covered lesions was higher (*p* < 0.001) when compared to all types of SLND, except for superextended SLND, which was comparable to ENRT. The authors concluded that limited or standard extended SLND might be insufficient as a salvage treatment approach and ENRT or superextended SLND should be preferred [33].

MDT to recurrent lymph node metastases is still controversial and there is an ongoing debate whether it definitely changes the disease outcome in the long-run or represents just “PSA cosmetics” that comes at a cost of potential toxicity [34]. Clearly, its oncologic benefit is dependent on patient selection. Fossati et al. evaluated 654 patients with nodal recurrence after RPE who underwent SLND: At multivariable analysis, Gleason grade group 5, time from RPE to PSA rising, ADT application at PSA rising after RPE, retroperitoneal or three or more spots at PET/CT scan and PSA level at SLND were significant predictors of clinical recurrence after SLND [35].

Ongoing clinical trials, such as OLIGOPELVIS (NCT02274779) that includes patients with 1–5 pelvic nodal oligometastases who are treated with high-dose radiotherapy and ADT for 6 months after prior radical prostate treatment and the PEACE V trial (NCT03569241) that randomizes patients with ≤3 oligorecurrent pelvic lymph node metastases between MDT with 6 months of ADT alone versus MDT with 6 months of ADT and whole pelvis RT will further help to optimize the therapeutic approach in patients with oligometastatic lymph node recurrences and are eagerly awaited.

Head-to-head comparisons of SLND to SLNRT, as was performed in the current study, give an insight of what might be the optimal treatment for patients with pelvic lymph node recurrences but are not without limitations. First, it has to be stated that the median follow-up was shorter for the SLND cohort. This might result in an underestimation of the real therapeutic effects of the two different treatment modalities. Second, this analysis was not performed as a matched pair analysis due to the small patient number. Third, bRFS is not the optimal endpoint as it is influenced by ADT use and 88% of SLNRT patients received ADT concomitantly with RT. This might lead to a more favourable bRFS in some patients, although the majority (50/67; 75%) had no ADT at last follow-up and 42/59 (71%) of those with ADT concomitantly to RT had discontinued it a median of 27 months (range 0–48 months) before last follow-up. Fourth, none of SLND patients has received postoperative adjuvant radiotherapy due to locally advanced tumour or positive surgical margins posing an undertreatment. This might partly explain the lower bRFS in SLND patients and might lead to the conclusion that SLND should primarily be undertaken in patients without locally advanced disease or positive surgical margins when adjuvant radiotherapy of prostatic fossa is withheld.

The aim of this comparative study was to explore the possible treatment options for patients with PSMA PET-positive, oligorecurrent nodal disease: With all its inherent flaws of such an retrospective analysis, SLNRT seems to be the preferred treatment option for patients with nodal recurrences after previous radical prostatectomy especially when undergoing a combination of SLNRT in the form of an ENRT as it is performed in our institution and concomitant ADT with overall an acceptable toxicity profile. The present results cannot be transferred to other surgical approaches like superextended SLND. Even though all patients received PSMA PET-guided MDT [36], one can state that with the current imaging modalities, also at the time of PSMA PET/CT a more restricted surgical or radiotherapy approach, like SBRT with the aim of less toxicity cannot be safely performed yet. Despite its limitations, the current study depicts real-world data from a tertiary-care reference centre and might be hypotheses-generating for future phase II trials.

## Conclusions

In the current study, rare comparative data from a contemporary patient cohort diagnosed within the PSMA-PET/CT era are provided. Significant differences regarding patient characteristics were observed that must be considered for future trial design. Overall, in this retrospective study a longer bRFS was observed in patients undergoing PSMA PET-informed SLNRT as compared to SLND for lymph node recurrent PCa. Findings need to be confirmed in a prospective randomized trial.

## Caption Electronic Supplementary Material

Supplemental Table 1: Univariate analysis of factors associated with biochemical recurrence-free survival after salvage lymph node dissection/salvage lymph node radiotherapy.
