# Supplementary material for: PSMA-positive nodal recurrence in prostate cancer: Salvage radiotherapy is superior to salvage lymph node dissection in retrospective analysis
Source: Strahlenther Onkol. 2020 Mar 24;196(7):637–46. doi: 10.1007/s00066-020-01605-z (PMC7305083; doi:10.1007/s00066-020-01605-z)
Supplement: Supplementary file 1 — Supplemental Table 1: Univariate analysis of factors associated with biochemical recurrence-free survival after salvage lymph node dissection/salvage lymph node radiotherapy. [file 66_2020_1605_MOESM1_ESM.docx]

**Supplemental Table 1:** Univariate analysis of factors associated with biochemical recurrence free survival after salvage lymph node dissection/salvage lymph node radiotherapy.

| Association with BRFS (<0.2ng/mL) | p-Value^a^ |
| --- | --- |
| SLND vs. SLNRT | 0.001 |
| GS ≤6-7 vs. GS 8-9 | 0.025 |
| T2 vs. T3-4 | 0.633 |
| N0/Nx vs. N1 | 0.834 |
| low/intermediate vs. high D’Amico | 0.515 |
| PSA at salvage therapy [ng/ml] | 0.001 |
| PSA persistence vs. PSA recurrence | 0.100 |
| PET-pos. LN pelvic vs. retroperitoneal/both | 0.217 |
| ᵃ chi-square test and Mann-Whitney U test, SLND: salvage lymph node dissection, SLNRT: salvage lymph node radiotherapy, BRFS: biochemical recurrence free survival, GS: Gleason Score; T: Tumor stage, N: Nodal stage, PSA: prostate specific antigen, LN: lymph node | |
